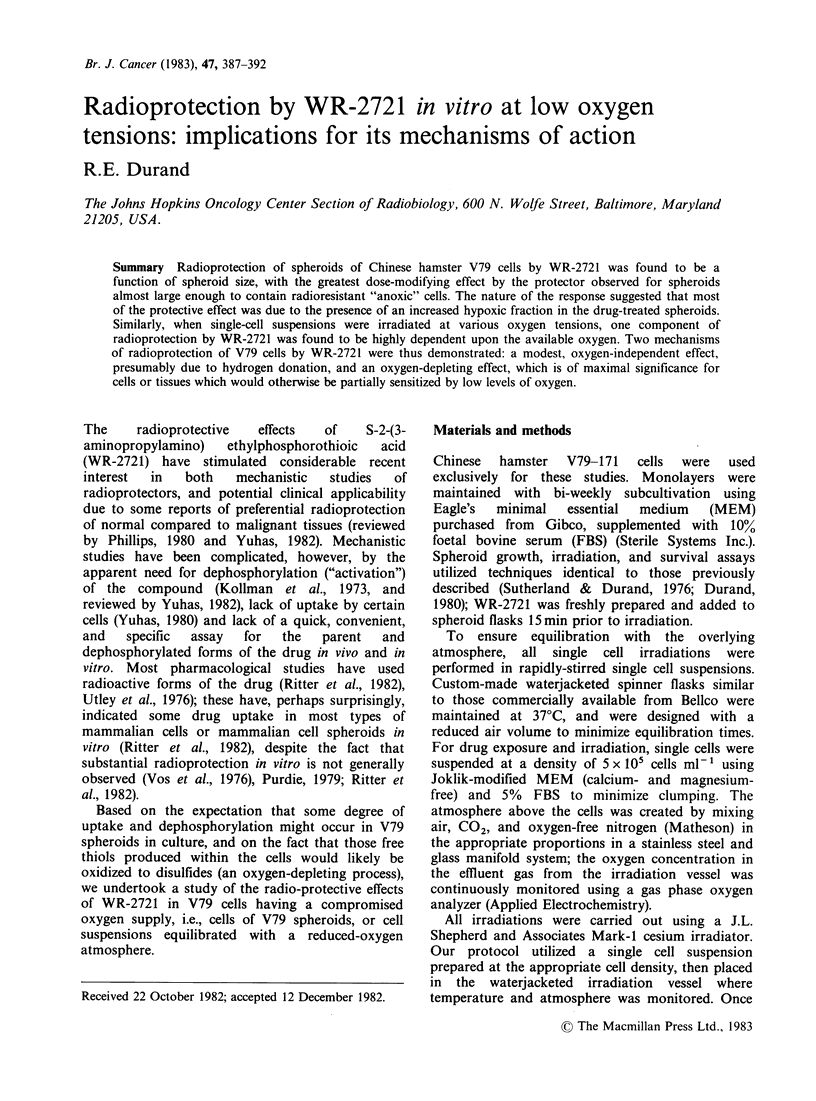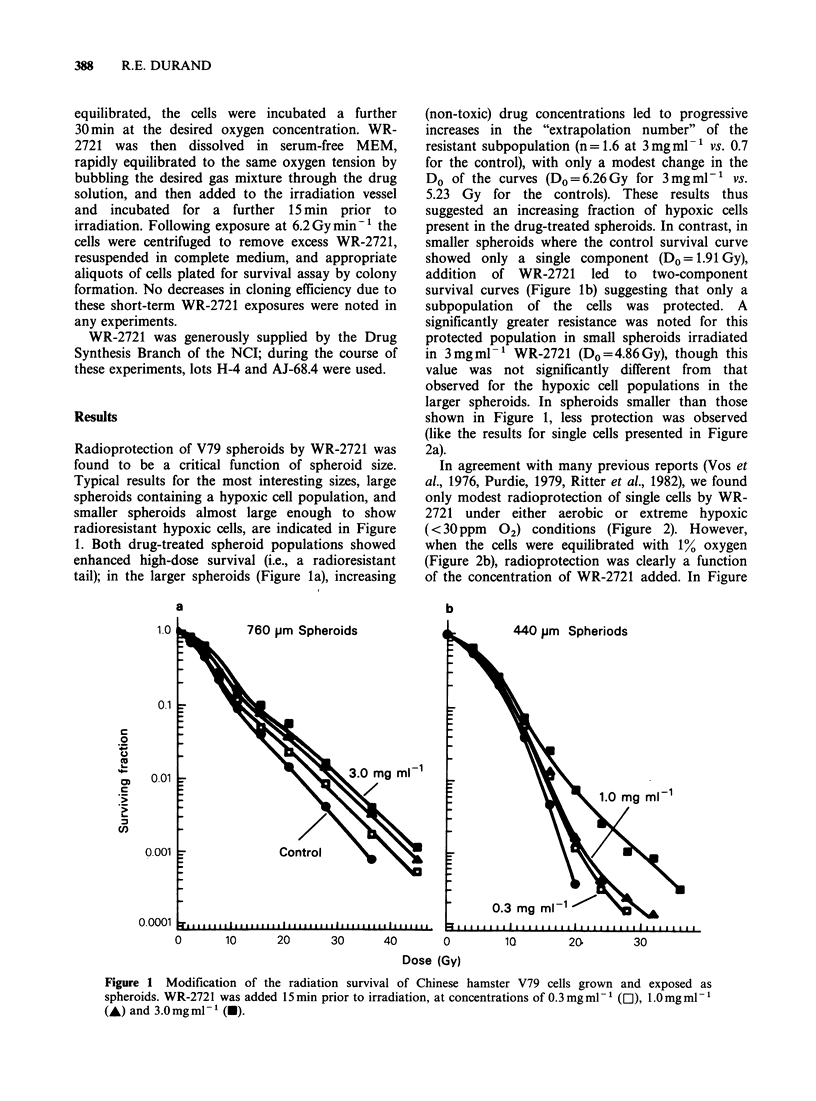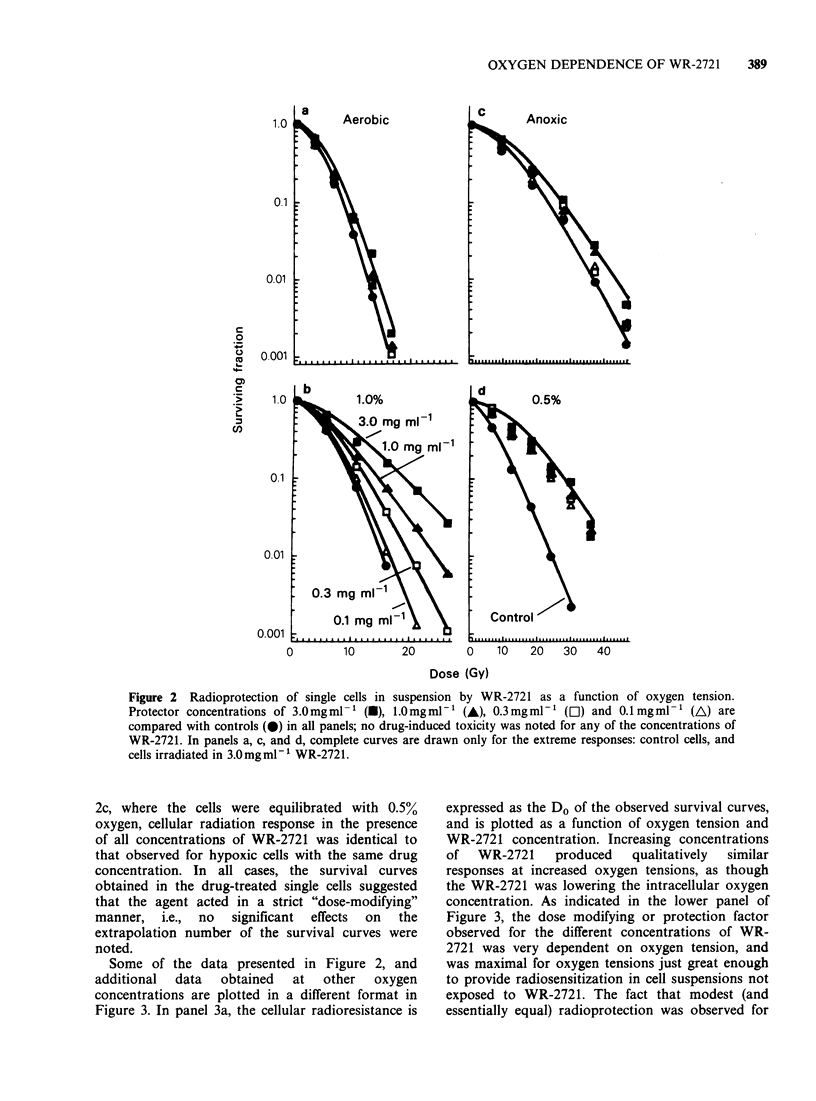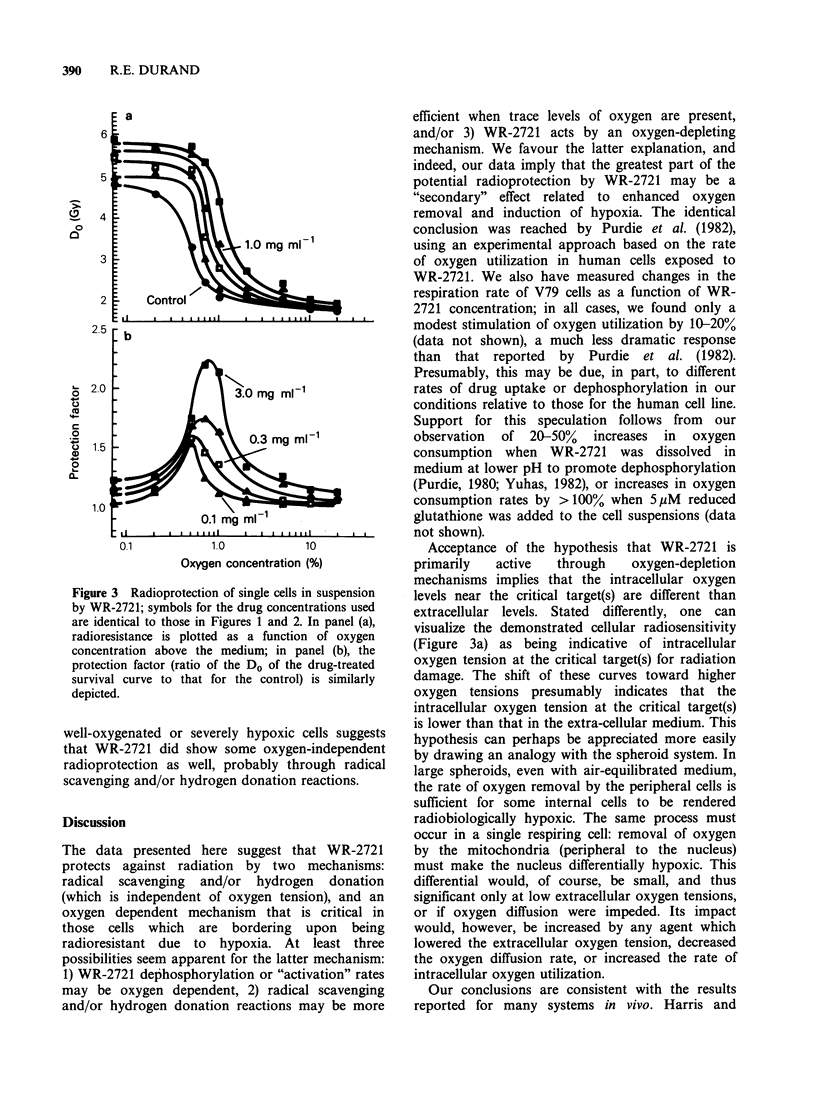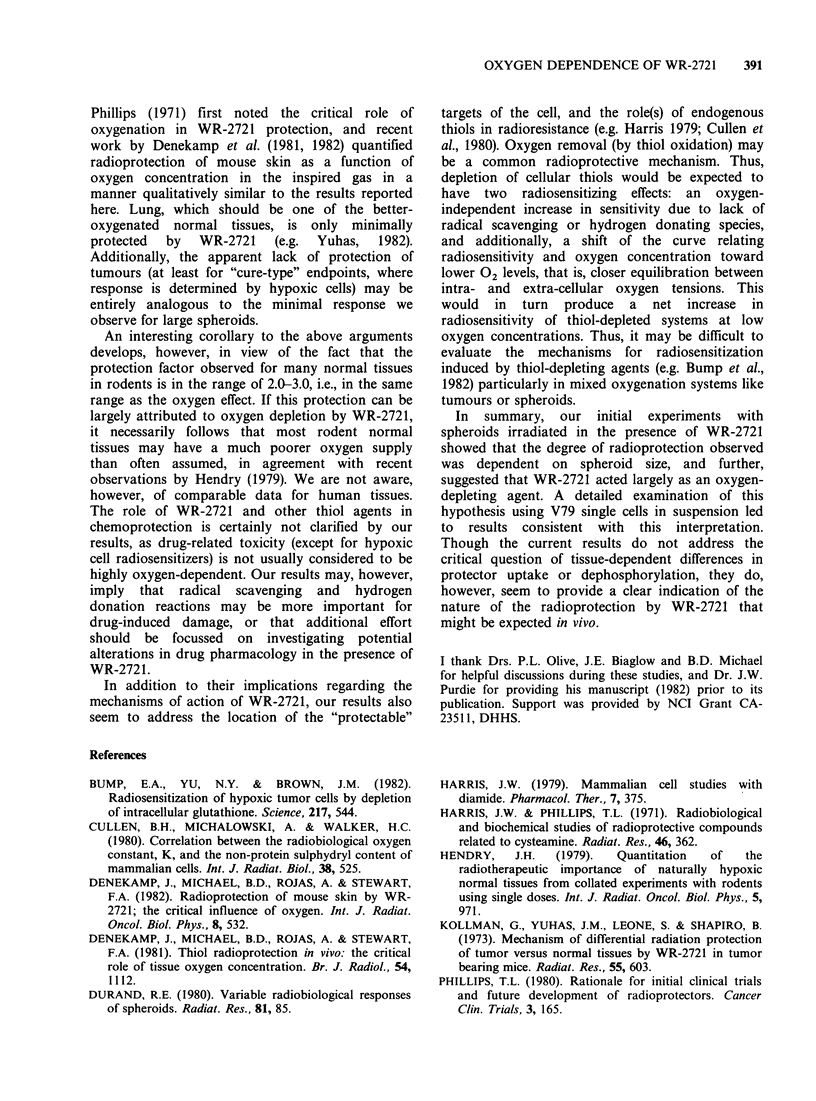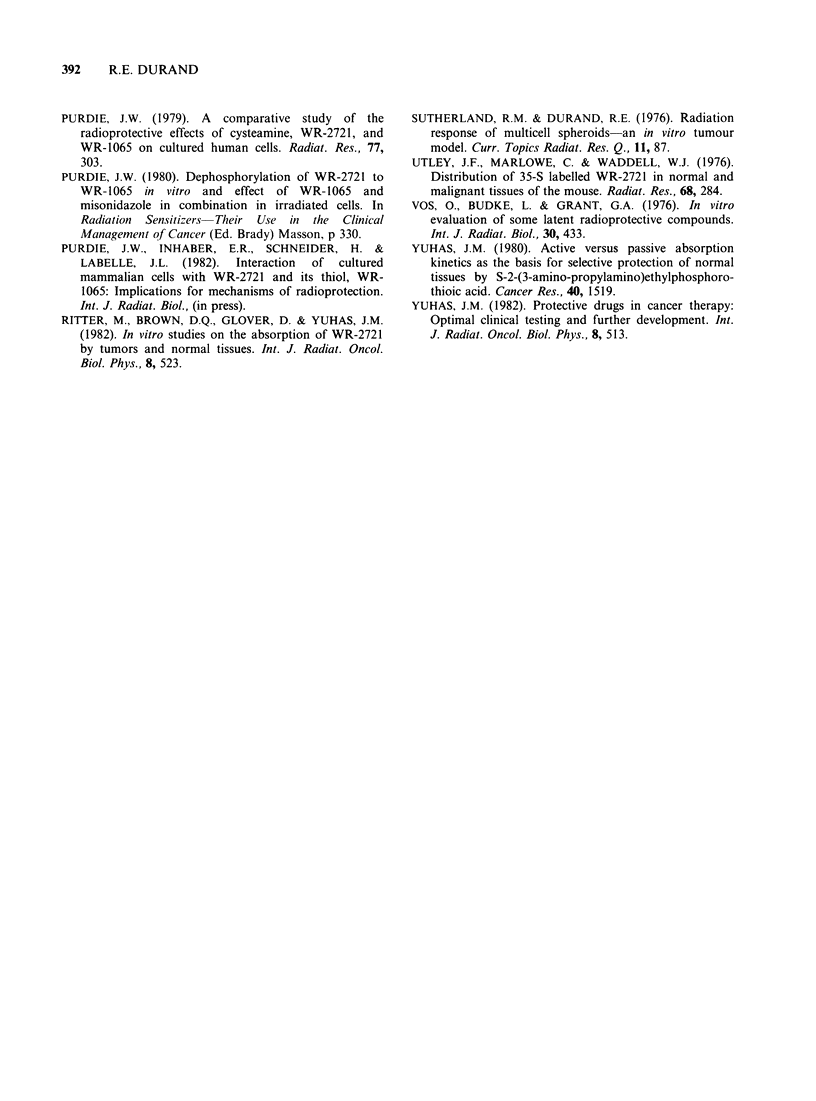# Radioprotection by WR-2721 in vitro at low oxygen tensions: implications for its mechanisms of action.

**DOI:** 10.1038/bjc.1983.58

**Published:** 1983-03

**Authors:** R. E. Durand

## Abstract

Radioprotection of spheroids of Chinese hamster V79 cells by WR-2721 was found to be a function of spheroid size, with the greatest dose-modifying effect by the protector observed for spheroids almost large enough to contain radioresistant "anoxic" cells. The nature of the response suggested that most of the protective effect was due to the presence of an increased hypoxic fraction in the drug-treated spheroids. Similarly, when single-cell suspensions were irradiated at various oxygen tensions, one component of radioprotection by WR-2721 was found to be highly dependent upon the available oxygen. Two mechanisms of radioprotection of V79 cells by WR-2721 were thus demonstrated: a modest, oxygen-independent effect, presumably due to hydrogen donation, and an oxygen-depleting effect, which is of maximal significance for cells or tissues which would otherwise be partially sensitized by low levels of oxygen.


*Br. J. Cancer* (1983), **47**, 387–392

### Radioprotection by WR-2721 *in vitro* at low oxygen tensions: implications for its mechanisms of action

R.E. Durand

*The Johns Hopkins Oncology Center Section of Radiobiology, 600 N. Wolfe Street, Baltimore, Maryland 21205, USA.*

**Summary** Radioprotection of spheroids of Chinese hamster V79 cells by WR-2721 was found to be a function of spheroid size, with the greatest dose-modifying effect by the protector observed for spheroids almost large enough to contain radioresistant "anoxic" cells. The nature of the response suggested that most of the protective effect was due to the presence of an increased hypoxic fraction in the drug-treated spheroids. Similarly, when single-cell suspensions were irradiated at various oxygen tensions, one component of radioprotection by WR-2721 was found to be highly dependent upon the available oxygen. Two mechanisms of radioprotection of V79 cells by WR-2721 were thus demonstrated: a modest, oxygen-independent effect, presumably due to hydrogen donation, and an oxygen-depleting effect, which is of maximal significance for cells or tissues which would otherwise be partially sensitized by low levels of oxygen.

The radioprotective effects of S-2-(3-aminopropylamino) ethylphosphorothioic acid (WR-2721) have stimulated considerable recent interest in both mechanistic studies of radioprotectors, and potential clinical applicability due to some reports of preferential radioprotection of normal compared to malignant tissues (reviewed by Phillips, 1980 and Yuhas, 1982). Mechanistic studies have been complicated, however, by the apparent need for dephosphorylation ("activation") of the compound (Kollman *et al*., 1973, and reviewed by Yuhas, 1982), lack of uptake by certain cells (Yuhas, 1980) and lack of a quick, convenient, and specific assay for the parent and dephosphorylated forms of the drug *in vivo* and *in vitro*. Most pharmacological studies have used radioactive forms of the drug (Ritter *et al*., 1982), Utley *et al*., 1976); these have, perhaps surprisingly, indicated some drug uptake in most types of mammalian cells or mammalian cell spheroids *in vitro* (Ritter *et al*., 1982), despite the fact that substantial radioprotection *in vitro* is not generally observed (Vos *et al*., 1976), Purdie, 1979; Ritter *et al*., 1982).

Based on the expectation that some degree of uptake and dephosphorylation might occur in V79 spheroids in culture, and on the fact that those free thiols produced within the cells would likely be oxidized to disulfides (an oxygen-depleting process), we undertook a study of the radio-protective effects of WR-2721 in V79 cells having a compromised oxygen supply, i.e., cells of V79 spheroids, or cell suspensions equilibrated with a reduced-oxygen atmosphere.

Received 22 October 1982; accepted 12 December 1982.

### Materials and methods

Chinese hamster V79–171 cells were used exclusively for these studies. Monolayers were maintained with bi-weekly subcultivation using Eagle's minimal essential medium (MEM) purchased from Gibco, supplemented with 10% foetal bovine serum (FBS) (Sterile Systems Inc.). Spheroid growth, irradiation, and survival assays utilized techniques identical to those previously described (Sutherland & Durand, 1976; Durand, 1980); WR-2721 was freshly prepared and added to spheroid flasks 15 min prior to irradiation.

To ensure equilibration with the overlying atmosphere, all single cell irradiations were performed in rapidly-stirred single cell suspensions. Custom-made waterjacketed spinner flasks similar to those commercially available from Bellco were maintained at 37°C, and were designed with a reduced air volume to minimize equilibration times. For drug exposure and irradiation, single cells were suspended at a density of $5\times10^5$ cells ml$^{-1}$ using Joklik-modified MEM (calcium- and magnesium-free) and 5% FBS to minimize clumping. The atmosphere above the cells was created by mixing air, $CO_2$, and oxygen-free nitrogen (Matheson) in the appropriate proportions in a stainless steel and glass manifold system; the oxygen concentration in the effluent gas from the irradiation vessel was continuously monitored using a gas phase oxygen analyzer (Applied Electrochemistry).

All irradiations were carried out using a J.L. Shepherd and Associates Mark-1 cesium irradiator. Our protocol utilized a single cell suspension prepared at the appropriate cell density, then placed in the waterjacketed irradiation vessel where temperature and atmosphere was monitored. Once

© The Macmillan Press Ltd., 1983

equilibrated, the cells were incubated a further 30 min at the desired oxygen concentration. WR-2721 was then dissolved in serum-free MEM, rapidly equilibrated to the same oxygen tension by bubbling the desired gas mixture through the drug solution, and then added to the irradiation vessel and incubated for a further 15 min prior to irradiation. Following exposure at $6.2\,Gy\,min^{-1}$ the cells were centrifuged to remove excess WR-2721, resuspended in complete medium, and appropriate aliquots of cells plated for survival assay by colony formation. No decreases in cloning efficiency due to these short-term WR-2721 exposures were noted in any experiments.

WR-2721 was generously supplied by the Drug Synthesis Branch of the NCI; during the course of these experiments, lots H-4 and AJ-68.4 were used.

### Results

Radioprotection of V79 spheroids by WR-2721 was found to be a critical function of spheroid size. Typical results for the most interesting sizes, large spheroids containing a hypoxic cell population, and smaller spheroids almost large enough to show radioresistant hypoxic cells, are indicated in Figure 1. Both drug-treated spheroid populations showed enhanced high-dose survival (i.e., a radioresistant tail); in the larger spheroids (Figure 1a), increasing (non-toxic) drug concentrations led to progressive increases in the "extrapolation number" of the resistant subpopulation ($n = 1.6$ at $3\,mg\,ml^{-1}$ *vs.* 0.7 for the control), with only a modest change in the $D_0$ of the curves ($D_0 = 6.26\,Gy$ for $3\,mg\,ml^{-1}$ *vs.* 5.23 Gy for the controls). These results thus suggested an increasing fraction of hypoxic cells present in the drug-treated spheroids. In contrast, in smaller spheroids where the control survival curve showed only a single component ($D_0 = 1.91\,Gy$), addition of WR-2721 led to two-component survival curves (Figure 1b) suggesting that only a subpopulation of the cells was protected. A significantly greater resistance was noted for this protected population in small spheroids irradiated in $3\,mg\,ml^{-1}$ WR-2721 ($D_0 = 4.86\,Gy$), though this value was not significantly different from that observed for the hypoxic cell populations in the larger spheroids. In spheroids smaller than those shown in Figure 1, less protection was observed (like the results for single cells presented in Figure 2a).

In agreement with many previous reports (Vos *et al.*, 1976, Purdie, 1979, Ritter *et al.*, 1982), we found only modest radioprotection of single cells by WR-2721 under either aerobic or extreme hypoxic (<30 ppm $O_2$) conditions (Figure 2). However, when the cells were equilibrated with 1% oxygen (Figure 2b), radioprotection was clearly a function of the concentration of WR-2721 added. In Figure

**Figure 1** Modification of the radiation survival of Chinese hamster V79 cells grown and exposed as spheroids. WR-2721 was added 15 min prior to irradiation, at concentrations of $0.3\,mg\,ml^{-1}$ (□), $1.0\,mg\,ml^{-1}$ (▲) and $3.0\,mg\,ml^{-1}$ (■).

**Figure 2** Radioprotection of single cells in suspension by WR-2721 as a function of oxygen tension. Protector concentrations of $3.0\,mg\,ml^{-1}$ (■), $1.0\,mg\,ml^{-1}$ (▲), $0.3\,mg\,ml^{-1}$ (□) and $0.1\,mg\,ml^{-1}$ (△) are compared with controls (●) in all panels; no drug-induced toxicity was noted for any of the concentrations of WR-2721. In panels a, c, and d, complete curves are drawn only for the extreme responses: control cells, and cells irradiated in $3.0\,mg\,ml^{-1}$ WR-2721.

2c, where the cells were equilibrated with 0.5% oxygen, cellular radiation response in the presence of all concentrations of WR-2721 was identical to that observed for hypoxic cells with the same drug concentration. In all cases, the survival curves obtained in the drug-treated single cells suggested that the agent acted in a strict "dose-modifying" manner, i.e., no significant effects on the extrapolation number of the survival curves were noted.

Some of the data presented in Figure 2, and additional data obtained at other oxygen concentrations are plotted in a different format in Figure 3. In panel 3a, the cellular radioresistance is expressed as the $D_0$ of the observed survival curves, and is plotted as a function of oxygen tension and WR-2721 concentration. Increasing concentrations of WR-2721 produced qualitatively similar responses at increased oxygen tensions, as though the WR-2721 was lowering the intracellular oxygen concentration. As indicated in the lower panel of Figure 3, the dose modifying or protection factor observed for the different concentrations of WR-2721 was very dependent on oxygen tension, and was maximal for oxygen tensions just great enough to provide radiosensitization in cell suspensions not exposed to WR-2721. The fact that modest (and essentially equal) radioprotection was observed for

**Figure 3** Radioprotection of single cells in suspension by WR-2721; symbols for the drug concentrations used are identical to those in Figures 1 and 2. In panel (a), radioresistance is plotted as a function of oxygen concentration above the medium; in panel (b), the protection factor (ratio of the $D_0$ of the drug-treated survival curve to that for the control) is similarly depicted.

well-oxygenated or severely hypoxic cells suggests that WR-2721 did show some oxygen-independent radioprotection as well, probably through radical scavenging and/or hydrogen donation reactions.

### Discussion

The data presented here suggest that WR-2721 protects against radiation by two mechanisms: radical scavenging and/or hydrogen donation (which is independent of oxygen tension), and an oxygen dependent mechanism that is critical in those cells which are bordering upon being radioresistant due to hypoxia. At least three possibilities seem apparent for the latter mechanism: 1) WR-2721 dephosphorylation or "activation" rates may be oxygen dependent, 2) radical scavenging and/or hydrogen donation reactions may be more efficient when trace levels of oxygen are present, and/or 3) WR-2721 acts by an oxygen-depleting mechanism. We favour the latter explanation, and indeed, our data imply that the greatest part of the potential radioprotection by WR-2721 may be a "secondary" effect related to enhanced oxygen removal and induction of hypoxia. The identical conclusion was reached by Purdie *et al.* (1982), using an experimental approach based on the rate of oxygen utilization in human cells exposed to WR-2721. We also have measured changes in the respiration rate of V79 cells as a function of WR-2721 concentration; in all cases, we found only a modest stimulation of oxygen utilization by 10–20% (data not shown), a much less dramatic response than that reported by Purdie *et al.* (1982). Presumably, this may be due, in part, to different rates of drug uptake or dephosphorylation in our conditions relative to those for the human cell line. Support for this speculation follows from our observation of 20–50% increases in oxygen consumption when WR-2721 was dissolved in medium at lower pH to promote dephosphorylation (Purdie, 1980; Yuhas, 1982), or increases in oxygen consumption rates by >100% when 5 $\mu$M reduced glutathione was added to the cell suspensions (data not shown).

Acceptance of the hypothesis that WR-2721 is primarily active through oxygen-depletion mechanisms implies that the intracellular oxygen levels near the critical target(s) are different than extracellular levels. Stated differently, one can visualize the demonstrated cellular radiosensitivity (Figure 3a) as being indicative of intracellular oxygen tension at the critical target(s) for radiation damage. The shift of these curves toward higher oxygen tensions presumably indicates that the intracellular oxygen tension at the critical target(s) is lower than that in the extra-cellular medium. This hypothesis can perhaps be appreciated more easily by drawing an analogy with the spheroid system. In large spheroids, even with air-equilibrated medium, the rate of oxygen removal by the peripheral cells is sufficient for some internal cells to be rendered radiobiologically hypoxic. The same process must occur in a single respiring cell: removal of oxygen by the mitochondria (peripheral to the nucleus) must make the nucleus differentially hypoxic. This differential would, of course, be small, and thus significant only at low extracellular oxygen tensions, or if oxygen diffusion were impeded. Its impact would, however, be increased by any agent which lowered the extracellular oxygen tension, decreased the oxygen diffusion rate, or increased the rate of intracellular oxygen utilization.

Our conclusions are consistent with the results reported for many systems *in vivo*. Harris and

Phillips (1971) first noted the critical role of oxygenation in WR-2721 protection, and recent work by Denekamp *et al.* (1981, 1982) quantified radioprotection of mouse skin as a function of oxygen concentration in the inspired gas in a manner qualitatively similar to the results reported here. Lung, which should be one of the better-oxygenated normal tissues, is only minimally protected by WR-2721 (e.g. Yuhas, 1982). Additionally, the apparent lack of protection of tumours (at least for "cure-type" endpoints, where response is determined by hypoxic cells) may be entirely analogous to the minimal response we observe for large spheroids.

An interesting corollary to the above arguments develops, however, in view of the fact that the protection factor observed for many normal tissues in rodents is in the range of 2.0–3.0, i.e., in the same range as the oxygen effect. If this protection can be largely attributed to oxygen depletion by WR-2721, it necessarily follows that most rodent normal tissues may have a much poorer oxygen supply than often assumed, in agreement with recent observations by Hendry (1979). We are not aware, however, of comparable data for human tissues. The role of WR-2721 and other thiol agents in chemoprotection is certainly not clarified by our results, as drug-related toxicity (except for hypoxic cell radiosensitizers) is not usually considered to be highly oxygen-dependent. Our results may, however, imply that radical scavenging and hydrogen donation reactions may be more important for drug-induced damage, or that additional effort should be focussed on investigating potential alterations in drug pharmacology in the presence of WR-2721.

In addition to their implications regarding the mechanisms of action of WR-2721, our results also seem to address the location of the "protectable" targets of the cell, and the role(s) of endogenous thiols in radioresistance (e.g. Harris 1979; Cullen *et al.*, 1980). Oxygen removal (by thiol oxidation) may be a common radioprotective mechanism. Thus, depletion of cellular thiols would be expected to have two radiosensitizing effects: an oxygen-independent increase in sensitivity due to lack of radical scavenging or hydrogen donating species, and additionally, a shift of the curve relating radiosensitivity and oxygen concentration toward lower $O_2$ levels, that is, closer equilibration between intra- and extra-cellular oxygen tensions. This would in turn produce a net increase in radiosensitivity of thiol-depleted systems at low oxygen concentrations. Thus, it may be difficult to evaluate the mechanisms for radiosensitization induced by thiol-depleting agents (e.g. Bump *et al.*, 1982) particularly in mixed oxygenation systems like tumours or spheroids.

In summary, our initial experiments with spheroids irradiated in the presence of WR-2721 showed that the degree of radioprotection observed was dependent on spheroid size, and further, suggested that WR-2721 acted largely as an oxygen-depleting agent. A detailed examination of this hypothesis using V79 single cells in suspension led to results consistent with this interpretation. Though the current results do not address the critical question of tissue-dependent differences in protector uptake or dephosphorylation, they do, however, seem to provide a clear indication of the nature of the radioprotection by WR-2721 that might be expected *in vivo*.

I thank Drs. P.L. Olive, J.E. Biaglow and B.D. Michael for helpful discussions during these studies, and Dr. J.W. Purdie for providing his manuscript (1982) prior to its publication. Support was provided by NCI Grant CA-23511, DHHS.

**References**

BUMP, E.A., YU, N.Y. & BROWN, J.M. (1982). Radiosensitization of hypoxic tumor cells by depletion of intracellular glutathione. *Science*, **217**, 544.

CULLEN, B.H., MICHALOWSKI, A. & WALKER, H.C. (1980). Correlation between the radiobiological oxygen constant, K, and the non-protein sulphydryl content of mammalian cells. *Int. J. Radiat. Biol.*, **38**, 525.

DENEKAMP, J., MICHAEL, B.D., ROJAS, A. & STEWART, F.A. (1982). Radioprotection of mouse skin by WR-2721; the critical influence of oxygen. *Int. J. Radiat. Oncol. Biol. Phys.*, **8**, 532.

DENEKAMP, J., MICHAEL, B.D., ROJAS, A. & STEWART, F.A. (1981). Thiol radioprotection *in vivo:* the critical role of tissue oxygen concentration. *Br. J. Radiol.*, **54**, 1112.

DURAND, R.E. (1980). Variable radiobiological responses of spheroids. *Radiat. Res.*, **81**, 85.

HARRIS, J.W. (1979). Mammalian cell studies with diamide. *Pharmacol. Ther.*, **7**, 375.

HARRIS, J.W. & PHILLIPS, T.L. (1971). Radiobiological and biochemical studies of radioprotective compounds related to cysteamine. *Radiat. Res.*, **46**, 362.

HENDRY, J.H. (1979). Quantitation of the radiotherapeutic importance of naturally hypoxic normal tissues from collated experiments with rodents using single doses. *Int. J. Radiat. Oncol. Biol. Phys.*, **5**, 971.

KOLLMAN, G., YUHAS, J.M., LEONE, S. & SHAPIRO, B. (1973). Mechanism of differential radiation protection of tumor versus normal tissues by WR-2721 in tumor bearing mice. *Radiat. Res.*, **55**, 603.

PHILLIPS, T.L. (1980). Rationale for initial clinical trials and future development of radioprotectors. *Cancer Clin. Trials*, **3**, 165.

PURDIE, J.W. (1979). A comparative study of the radioprotective effects of cysteamine, WR-2721, and WR-1065 on cultured human cells. *Radiat. Res.*, **77**, 303.

PURDIE, J.W. (1980). Dephosphorylation of WR-2721 to WR-1065 *in vitro* and effect of WR-1065 and misonidazole in combination in irradiated cells. In *Radiation Sensitizers—Their Use in the Clinical Management of Cancer* (Ed. Brady) Masson, p 330.

PURDIE, J.W., INHABER, E.R., SCHNEIDER, H. & LABELLE, J.L. (1982). Interaction of cultured mammalian cells with WR-2721 and its thiol, WR-1065: Implications for mechanisms of radioprotection. *Int. J. Radiat. Biol.*, (in press).

RITTER, M., BROWN, D.Q., GLOVER, D. & YUHAS, J.M. (1982). *In vitro* studies on the absorption of WR-2721 by tumors and normal tissues. *Int. J. Radiat. Oncol. Biol. Phys.*, **8**, 523.

SUTHERLAND, R.M. & DURAND, R.E. (1976). Radiation response of multicell spheroids—an *in vitro* tumour model. *Curr. Topics Radiat. Res. Q.*, **11**, 87.

UTLEY, J.F., MARLOWE, C. & WADDELL, W.J. (1976). Distribution of 35-S labelled WR-2721 in normal and malignant tissues of the mouse. *Radiat. Res.*, **68**, 284.

VOS, O., BUDKE, L. & GRANT, G.A. (1976). *In vitro* evaluation of some latent radioprotective compounds. *Int. J. Radiat. Biol.*, **30**, 433.

YUHAS, J.M. (1980). Active versus passive absorption kinetics as the basis for selective protection of normal tissues by S-2-(3-amino-propylamino)ethylphosphorothioic acid. *Cancer Res.*, **40**, 1519.

YUHAS, J.M. (1982). Protective drugs in cancer therapy: Optimal clinical testing and further development. *Int. J. Radiat. Oncol. Biol. Phys.*, **8**, 513.